# Ileus Due to Iron Pills: A Case Report and Literature Report on the Importance of Stool Softeners

**DOI:** 10.7759/cureus.8392

**Published:** 2020-06-01

**Authors:** Swetha Parvataneni, Min Maw

**Affiliations:** 1 Internal Medicine, Geisinger Health System, Lewistown, USA; 2 Hospital Medicine, Geisinger Lewistown Hospital, Lewistown, USA

**Keywords:** iron tablets, ileus, bowel obstruction

## Abstract

Iron deficiency anemia is the leading cause of anemia in the U.S. and throughout the world. The most commonly available over-the-counter treatment option is oral iron supplements. As they are easily available and inexpensive, the use of iron supplements has increased alongside the increased prevalence of iron deficiency anemia. However, iron pills cause various side effects such as nausea, vomiting, constipation, and, rarely, bowel perforation. Iron pills causing ileus secondary to bowel obstruction were rarely reported. Here, we present the case of a female patient with symptoms of bowel obstruction, without any predisposing surgical history, diagnosed with ileus secondary to bowel obstruction from constipation on imaging. In this case discussion and review, we provide a detailed discussion on iron and its gastrointestinal (GI) pathophysiology, bowel obstruction and its causes, associated mortality rates, and management.

## Introduction

Bowel obstruction is characterized by either partial or complete blockage of the passage of intestinal contents through the small or large bowel. In the U.S., bowel obstruction occurs at an incidence of 350,000 per annum [1]. Small bowel obstruction carries associated morbidity and mortality rates of 13.8% and 3.4%, respectively, with higher rates reported for large colonic bowel obstructions [2]. The causes of small bowel obstruction include adhesions, hernia, neoplasms, and Crohn’s disease. Among all causes, adhesions, hernia, and neoplasms contribute to ~89% of cases [3]. Cancer contributes to the majority of large bowel obstructions, followed by volvulus and diverticulitis. A 2016 study reported approximately 107,603 hospital admissions with bowel obstruction secondary to adhesions; given the primary cause, approximately 205,712 major emergency adhesiolysis procedures are performed each year in the U.S. [4]. Patients with bowel obstruction often present with abdominal pain, nausea, vomiting, abdominal distension, and constipation. Rarely, these cases can progress to symptoms of ileus, bowel infarction, and perforation, depending on the degree of bowel obstruction [5]. In addition to the above-mentioned causes, drugs such as opiates are known to cause bowel obstruction, but cases of iron pills causing ileus due to bowel obstruction from constipation have rarely been reported. From the literature, we know that iron pills have significant gastrointestinal (GI) side-effects, but no cases have been reported of ileus secondary to bowel obstruction without past surgical history. Here, we present an interesting case of a female patient who was on iron pills for recently diagnosed iron deficiency anemia, with no significant past surgical history, who presented with ileus secondary to bowel obstruction due to constipation.

## Case presentation

A 55-year-old female with a past medical history of back pain and use of Tylenol, along with depression, anxiety, and hypertension, presented to the emergency room with complaints of abdominal pain for one week. Her symptoms started with intermittent, self-resolving abdominal pain, which gradually progressed to constant pain of 10/10 intensity that radiated down to approximately the umbilical area. She was nauseous but had not been vomiting. The patient reported that, prior to the week of symptoms, she had started taking iron pills and had not been feeling well since then. She had been constipated and unable to have a bowel movement for the prior week, reporting that her stool is "hard as a rock" and was only able to defecate small amounts, which is unusual for her. She had no associated symptoms of fevers or chills and no reported past surgical history.

**Figure 1:**
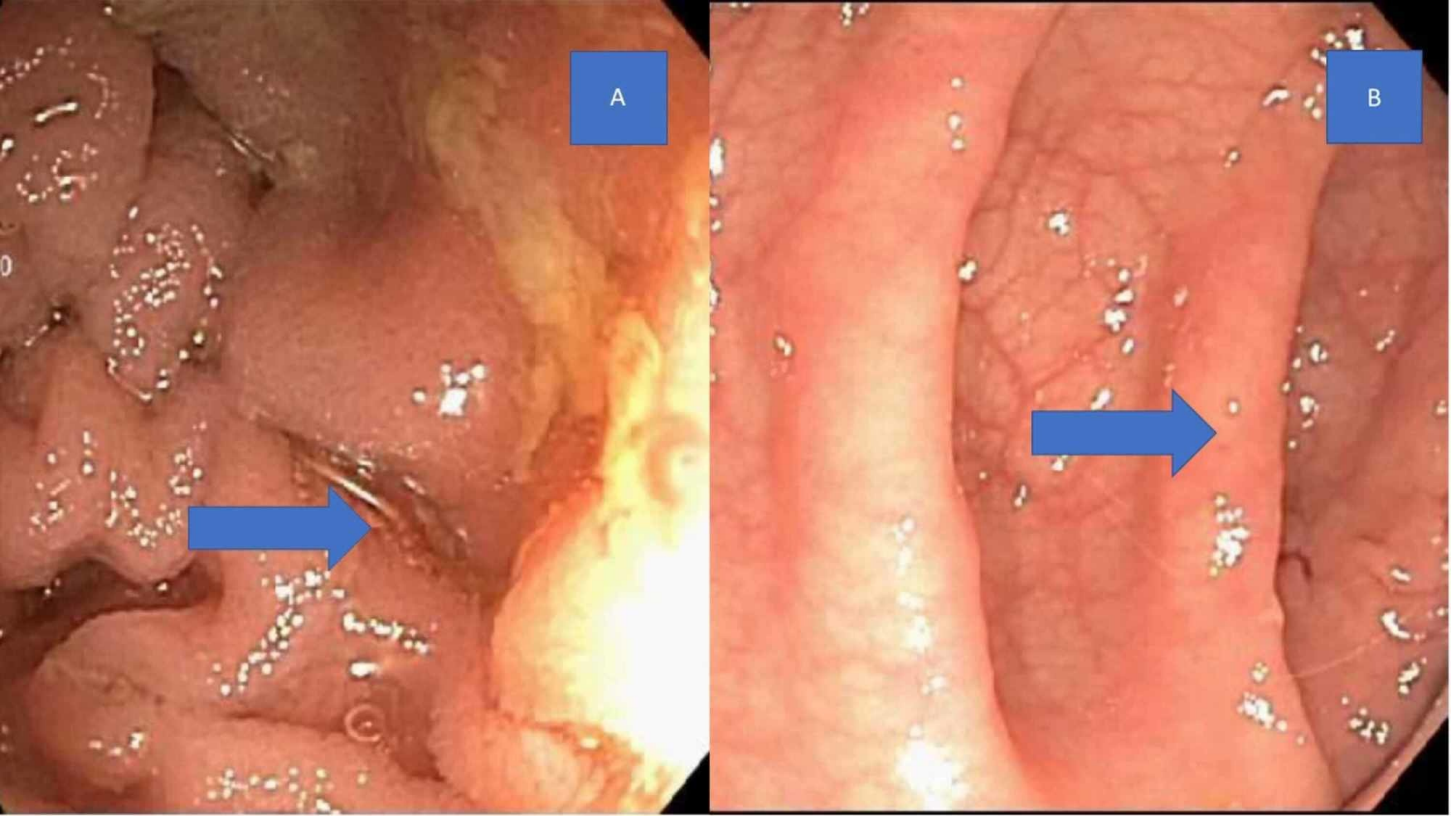
Colonoscopy 1A: Normal terminal ileum (shown by arrow), 1B: Normal descending colon (shown by arrow)

**Figure 2:**
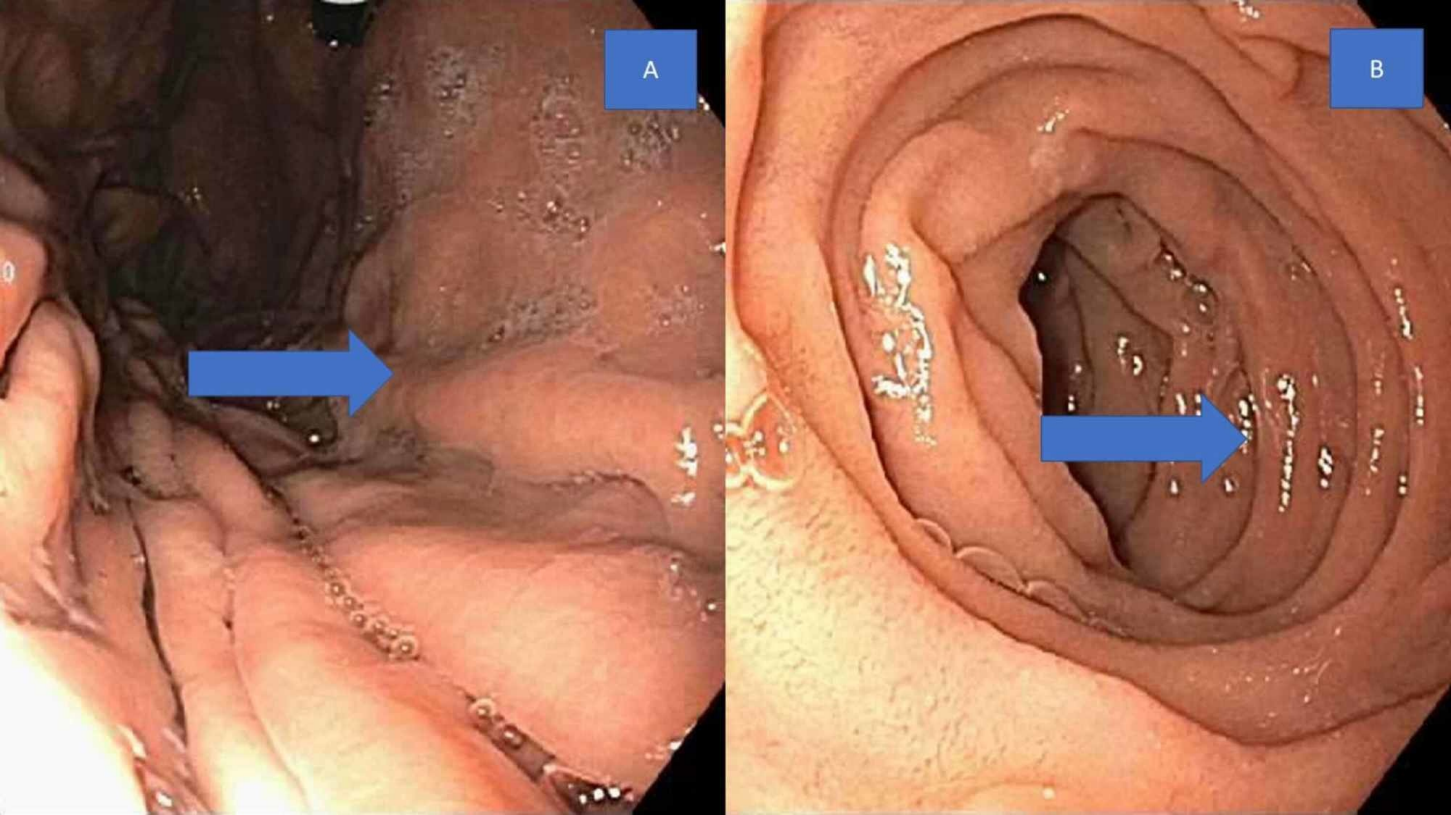
Endoscopy 2A: Normal gastric fundus (as shown by arrow), 2B: Normal second part of the duodenum (as shown by arrow)

On admission, the patient’s vitals were stable with a heart rate (HR) of 75, blood pressure (BP) of 138/70, temperature of 36.8 °C, and respiratory rate of 20 per minute. Her labs showed leukocytosis with a white cell count of 14,000 and anemia with hemoglobin of 8.6. She had recently received a colonoscopy (Figure 1) and endoscopy (Figure 2) for anemia and heme positive stools; both were normal.

**Figure 3:**
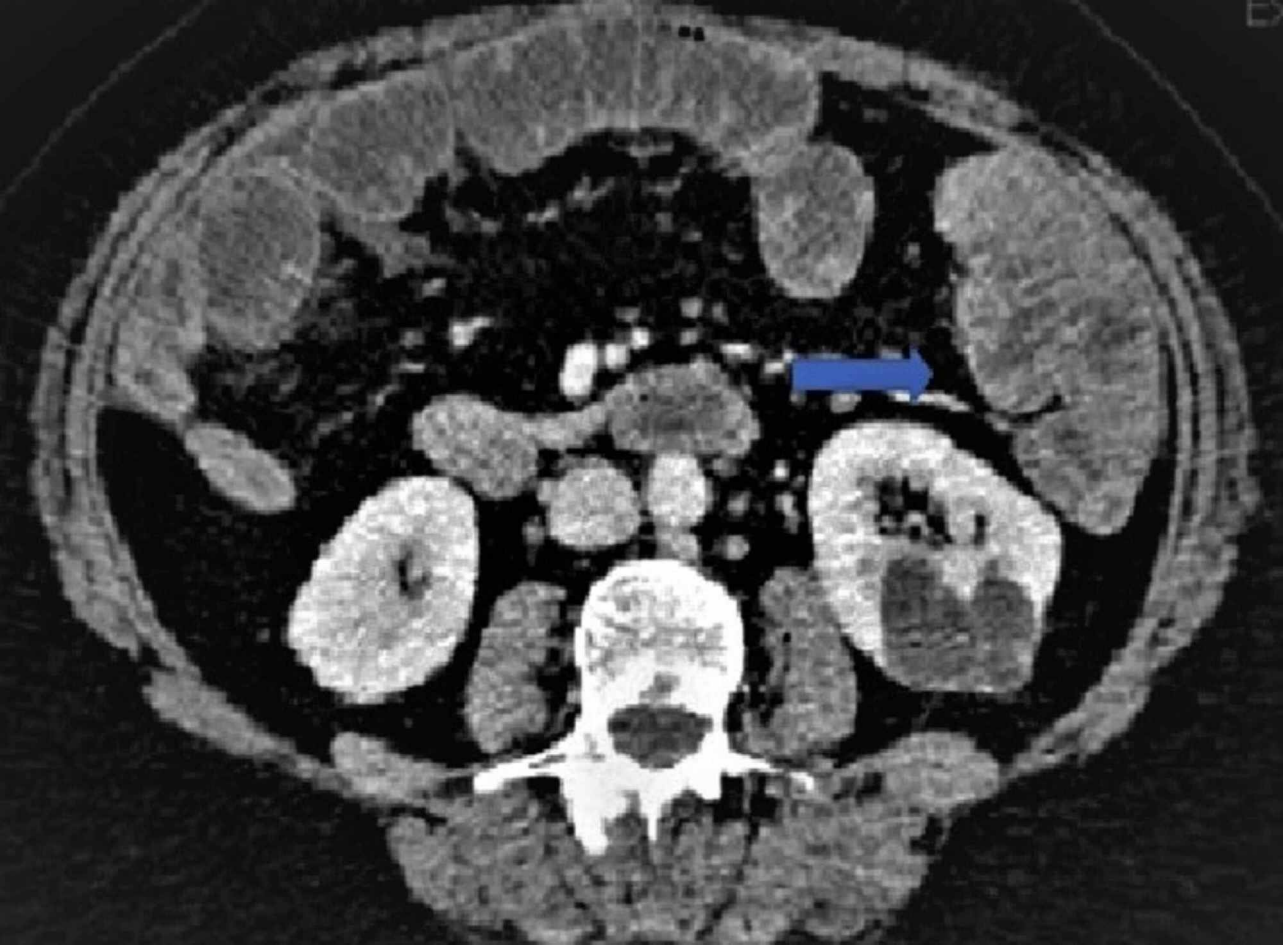
CT scan Large stool burden in the colon (shown by arrow) CT: computed tomography

Computed tomography (CT) abdomen and pelvis showed multiple fluid-filled loops of the small bowel, representing the ileus and large stool burden (Figure 3).

**Figure 4:**
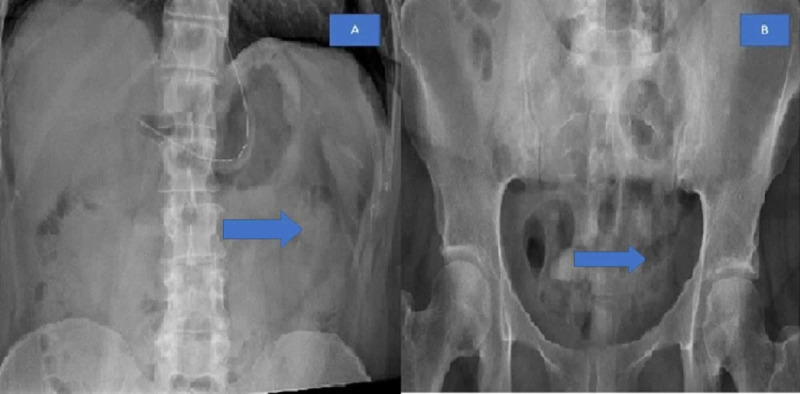
X-ray 4A, 4B: Repeat X-ray abdomen in 24 hours shows complete resolution of bowel obstruction and normal gas pattern (shown by arrows)

General surgery was consulted, and a nasogastric tube was placed. The patient was managed with intravenous (IV) fluids and was kept nil by mouth. The pain was managed with intravenous Tylenol. Serial abdominal exams were performed during the stay. After 24 hours, the patient began passing flatus, and at 48 hours after admission, the patient had multiple bowel movements. Repeat X-ray abdomen showed a normal bowel pattern, and her white cell count decreased to 7,000 (Figure 4).

Her gastric tube was removed, and she was started on a full liquid diet that was slowly advanced to a regular fiber diet. The patient was re-started on iron pills, with detailed education on fiber intake, stool softeners, and the potential side effects of iron pills, and discharged home in stable condition.

## Discussion

Anemia is the most common healthcare-related issue worldwide, with an estimated prevalence of 32.9% in 2010 [6]. Iron deficiency is listed as the leading cause of anemia, consistent with a 2013 report on the global burden of disease injuries and risk factors [7]. In the U.S., approximately 10 million people are iron deficient and approximately 5 million people suffer from iron deficiency anemia [8]. Data from national health and nutrition examination surveys from 2003 to 2012 evaluated the prevalence rate of anemia and moderate-severe anemia in the U.S. The data revealed an increased prevalence of 7.1% and 1.9% over the 10-year period, and Hispanics, non-Hispanic blacks, pregnant women and women overall were at increased risk, with peaks reported in the age groups of 40-49 years and 80-85 years [9]. Common causes included pregnancy, menstrual phase in the reproductive age group, peptic ulcer disease, neoplasm, and dietary deficiency. The prevalence and causes of iron deficiency anemia vary according to the geographic area [10].

In addition to dietary intake, the proposed treatment for iron deficiency anemia is oral iron supplementation. Various oral, over-the-counter formulations are available in the market, such as ferrous sulfate, ferrous fumarate, and ferrous gluconate. The daily recommended dose of iron is 100 to 200 mg of elemental iron, but studies have shown successful management with low daily doses of 15 to 20 mg [11-13]. Oral iron supplementation is effective in patients with an intact intestinal epithelium. In inflamed GI diseases, such as celiac disease and inflammatory bowel disease, for adequate absorption, IV iron is more effective as compared to the oral formulation. In other normal subjects with an intact intestinal epithelium, it is very important to know when to switch from oral to intravenous administration; a study published in 2017 analyzed five randomized trials and proposed IV treatment when iron levels fail to rise above 1.0 g /dL on day 14 in patients on oral iron supplementation [14]. Intravenous iron was found to be more effective as compared to the oral form in iron deficiency anemia patients; however, its use is limited by the need for administration by a healthcare worker and comparatively greater expensive to oral formulations [15]. The most common side effects associated with iron supplementation are GI-related events such as constipation, nausea, and diarrhea. Fatal bowel perforation has been occasionally reported in children due to iron poisoning, similar to our patient who developed ileus and bowel obstruction from iron pills [16].

Here we discuss the mechanism of action of iron and its absorption through the GI wall. Iron is an essential trace element for hemopoiesis that is available either in ferric (Fe3+) or ferrous (Fe2+) form; the ferrous form is very soluble and easily absorbed. Located in the acidic environment of duodenal cell walls, cytochrome b protein is a ferrireductase enzyme that converts ferric iron to its ferrous form. The ferrous form is then transported into enterocytes via the divalent metal transporter. This proton-coupled transporter causes the influx of ferrous into cells and the efflux of protons into the intestinal lumen. Normally, these protons are recycled elsewhere in the lumen via the sodium-hydrogen exchanger. The mechanism by which iron causes constipation is unclear, but it is thought that excess iron ions in the stomach facilitate the transport of water into the intestinal lumen by an osmotic gradient. This water is pulled from the lower GI system to maintain the acid-base balance throughout the GI system, which results in stool hardening and constipation. If not addressed in a timely manner, patients can present to the hospital with symptoms of ileus due to bowel obstruction from constipation, as evident in our case [17-18].

Imaging modalities, such as X-ray and CT scan with contrast are used in the diagnosis of small bowel obstruction. Depending on the symptoms and imaging findings, patients are managed either medically or surgically. If the patient is stable and there is no evidence of free intraperitoneal fluid or mesenteric edema and no signs of ischemia on imaging, the patient can be managed conservatively. Initial medical management includes fluid resuscitation, nil by mouth, nasogastric tube, and serial abdominal examinations to monitor impending signs of ischemia/ perforation. When the patient can pass gas or their symptoms improve, the nasogastric tube is removed, and the diet is advanced slowly. In addition, a trial of gastrograffin reported symptom improvement in 81.55% of patients over a short time period. If there is no response to a conservative approach in 48 hours, repeat imaging is conducted, and surgery may be recommended based on the findings [19-20]. Our patient responded well to a conservative approach, her diet was resumed, and she was discharged in stable condition after four days. At the time of discharge, she was provided with education on dietary fiber intake and stool softener use with iron pills.

## Conclusions

In conclusion, iron deficiency is one of the leading causes of anemia and a health-related burden in the U.S. Being one of the leading causes of anemia and with its increased incidence, there has been an increased use of over-the-counter iron pills without appropriate evaluation and discussion with the physicians. Iron pills have significant side effects on the GI system. Patients often refuse to take them because of the worrisome side effects. It is very important for the physician to educate the patients on these side effects and the necessary preventive measures.
